# A randomized, placebo-controlled clinical trial evaluating the safety and efficacy of the once-weekly DPP-4 inhibitor omarigliptin in patients with type 2 diabetes mellitus inadequately controlled by glimepiride and metformin

**DOI:** 10.1186/s12902-017-0219-x

**Published:** 2017-11-06

**Authors:** Seung-Hwan Lee, Ira Gantz, Elizabeth Round, Melanie Latham, Edward A. O’Neill, Paulette Ceesay, Shailaja Suryawanshi, Keith D. Kaufman, Samuel S. Engel, Eseng Lai

**Affiliations:** 1 Department of Internal Medicine, Division of Endocrinology and Metabolism, Seoul St.Mary’s Hospital, College of Medicine, The Catholic University of Korea, Seoul, Republic of Korea; 2 Merck & Co., Inc., 2000 Galloping Hill Road, Kenilworth, NJ 07033 USA

**Keywords:** MK-3102, Incretin therapy, Oral antihyperglycemic agent

## Abstract

**Background:**

Type 2 diabetes (T2D) is a progressive disease that often requires a patient to use multiple antihyperglycemic agents to achieve glycemic control with disease progression. Omarigliptin is a once-weekly dipeptidyl peptidase-4 inhibitor. The purpose of this trial was to assess the efficacy and safety of adding omarigliptin to the treatment regimen of patients with T2D inadequately controlled by dual therapy with metformin and glimepiride.

**Methods:**

Patients with T2D and HbA1c ≥7.5% and ≤10.5% while on metformin (≥1500 mg/day) and glimepiride (≥4 mg/day) were randomized to omarigliptin 25 mg once-weekly (*N* = 154) or placebo (*N* = 153) for 24 weeks. The primary objective was to assess whether omarigliptin was superior to placebo in reducing HbA1c at Week 24. Secondary objectives were to assess the effects of omarigliptin vs. placebo on FPG and the proportion of subjects attaining HbA1c goals of <7% and <6.5%.

**Results:**

From a mean baseline HbA1c of 8.5% (omarigliptin) and 8.6% (placebo), the least squares (LS) mean change from baseline in HbA1c at Week 24 was −0.67% in the omarigliptin group and −0.06% in the placebo group, with a between-group difference (95% CI) of −0.61% (−0.85, −0.38). Treatment with omarigliptin resulted in a significantly greater reduction in FPG relative to placebo (LS mean difference [95% CI] -0.9 mmol/L [−1.4, −0.4]; *p* < 0.001). The proportion of patients achieving glycemic goals of <7.0% and <6.5% was higher in the omarigliptin group relative to the placebo group. The overall incidences of adverse events (AEs), serious AEs, drug-related AEs and discontinuations were generally similar between treatment groups. The incidence of symptomatic hypoglycemia was 10.5% in the omarigliptin group and 8.5% in the placebo group. Relative to baseline, omarigliptin and placebo treatments were associated with LS mean changes in body weight of −0.1 kg and −0.9 kg, respectively.

**Conclusion:**

In patients with T2D and inadequate glycemic control on dual therapy with metformin and glimepiride, compared with placebo, once-weekly omarigliptin provided greater improvement in glycemic control and was generally well tolerated.

**Trial registration:**

ClinicalTrials.gov: NCT01704261, EudraCT Number: 2012-002612-10. Trial Registration Date: October 8, 2012.

## Background

Type 2 diabetes (T2D) is a progressive disease and most patients eventually require treatment with multiple antihyperglycemic agents in order to attain and remain within glycemic goals. One commonly used strategy for the treatment of T2D over time is initial monotherapy with the biguanide metformin [1], which lowers hepatic glucose production, followed by the addition of a sulfonylurea, a class of antihyperglycemic agent which mediates glucose-independent insulin secretion [2]. If additional glycemic control is required, patients can be advanced to triple oral therapy by adding a dipeptidyl peptidase-4 (DPP-4) inhibitor, a class of antihyperglycemic agent which stabilizes incretin peptides (e.g., glucagon-like peptide 1 and glucose-dependent insulinotropic peptide), thus enhancing glucose-dependent insulin secretion [3].

Omarigliptin (MK-3102) is an oral DPP-4 inhibitor with a half-life that enables once-weekly (q.w.) dosing [4] that is approved in Japan. Herein we report the results of a clinical study that compared the glycemic efficacy and safety of omarigliptin 25 mg administered q.w. with placebo when added to treatment of patients with inadequate glycemic control on the combination of metformin and glimepiride.

## Methods

### Patients

Eligible patients were ≥18 years of age with T2D and an HbA1c ≥7.5% and ≤10.5% on dual combination therapy with metformin ≥1500 mg/day for ≥12 weeks and either glimepiride or another sulfonylurea (see details below related to up-titration of glimepiride or switch to glimepiride).

Patients were excluded from the study if they had type 1 diabetes, a history of ketoacidosis, active liver disease, new or worsening signs or symptoms of coronary heart disease or congestive heart failure within the past 3 months, a history of malignancy or hematological disorders, or if they had been treated with any antihyperglycemic agent other than the protocol-required metformin and sulfonylurea within 12 weeks prior to signing informed consent. Laboratory exclusion criteria included serum alanine aminotransferase or aspartate aminotransferase levels >2 times the upper limit of normal (ULN), triglycerides >6.8 mmol/L or thyroid-stimulating hormone outside the central laboratory normal range. Due to the use of metformin in the study (and varying recommendations for its use among countries), patients with estimated glomerular filtration rate (based on modification of diet in renal disease formula [5]) <60 mL/min/1.73 m^2^, or creatinine ≥123.8 μmol/L (males) or ≥114.9 μmol/L (females) were also excluded.

### Study design

**Fig. 1:**
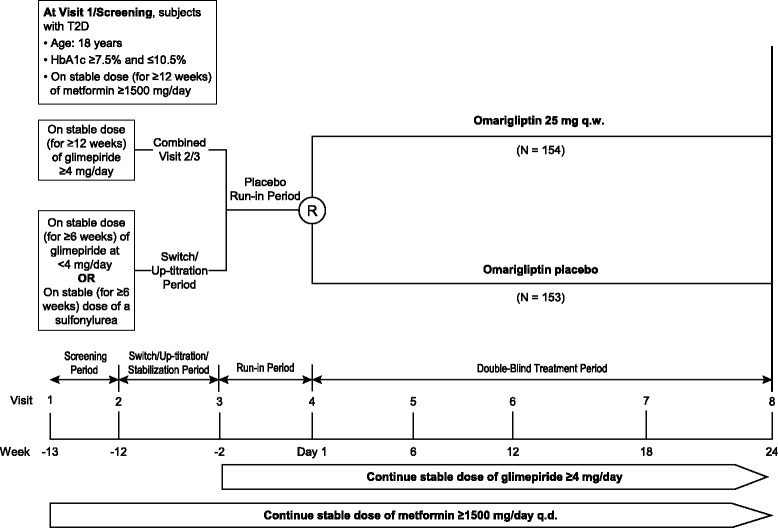
Study design; T2D = type 2 diabetes mellitus; q.w. = once weekly; R = randomization; q.d. = once daily

This was a multinational, randomized, placebo-controlled, double-blind study conducted at 51 sites in 6 countries (15 in the United States, 14 in Romania, 7 in South Africa, 6 in Poland, 5 in the Republic of Korea, and 4 in Russia). The study duration was up to 40 weeks and included a 1-week screening period, a sulfonylurea switch (for subjects not on glimepiride) and a glimepiride up-titration period (for subjects on <4 mg/day of glimepiride) of up to 4 weeks, followed by a 6-week dose-stabilization period (after which glimepiride was not to be further up-titrated), a 2-week single-blind placebo run-in period, a 24-week double-blind treatment period, and a telephone contact 21 days after the last dose of blinded study medication (omarigliptin/matching placebo) (Fig. 1).

Patients who were treated with a stable dose of metformin ≥1500 mg/day and glimepiride ≥4 mg/day for ≥12 weeks and who met all other entry criteria directly entered a 2-week single-blind placebo run-in. Patients on metformin ≥1500 mg/day for ≥12 weeks, but on glimepiride <4 mg/day or a sulfonylurea other than glimepiride for ≥6 weeks entered a 1-4-week study period during which glimepiride was up-titrated to ≥4 mg/day or patients were switched from the other sulfonylurea to glimepiride ≥4 mg/day. This 1-4 week period was followed by a 6-week dose stabilization period before entering the 2-week single blind placebo run-in. At the beginning of the run-in period, patients who had up-titration of their glimepiride to ≥4 mg/day or who were switched to glimepiride ≥4 mg/day were required to have an HbA1c ≥7.5% and ≤10.5%. At randomization, all patients were required to have a fasting finger-stick glucose >7.0 mmol/L and <14.4 mmol/L.

After the run-in period, patients were randomized centrally, using an interactive voice response system, in a 1:1 ratio to omarigliptin 25 mg q.w. or matching placebo. Randomization was stratified based on sulfonylurea status at screening: (1) glimepiride ≥4 mg/day; (2) glimepiride <4 mg/day; and (3) a sulfonylurea other than glimepiride.

After randomization, patients were to remain on their stable dose of metformin (≥1500 mg/day) and glimepiride (≥4 mg/day). However, the dose of glimepiride could be down-titrated for hypoglycemia to a minimum dose of 1 mg/day.

Patient compliance with omarigliptin (omarigliptin matching placebo) was assessed by site pill count at each visit during the treatment period. Compliance was defined as (Number of Compliant Days)/(Number of Days in the Double-blind Treatment Period) × 100%.

Patients who did not meet progressively stricter prespecified glycemic control criteria post-randomization (from Day 1 through Week 6, fasting plasma glucose [FPG] > 14.99 mmol/L; after Week 6 through Week 12, FPG > 13.32 mmol/L; after Week 12 through Week 24 FPG > 11.10 mmol/L) were discontinued from the study.

The study (MK-3102-022; NCT01704261, EudraCT: 2012-002612-10), registered October 8, 2012, was conducted in accordance with the principles of Good Clinical Practice and was approved by the appropriate institutional review boards and regulatory agencies. Written informed consent was obtained from all study participants.

### Study evaluations

The primary objectives of this study were to assess the safety and tolerability of omarigliptin and to compare its efficacy with that of placebo after 24 weeks of treatment. The primary hypothesis of the study was that addition of treatment with omarigliptin provides greater reduction in HbA1c compared with the addition of placebo.

Secondary objectives were to assess the effect of the addition of omarigliptin compared with placebo on FPG and on the percentage of patients with HbA1c of <7.0% and <6.5%, after 24 weeks of treatment.

### Efficacy endpoints

Efficacy endpoints were changes from baseline in HbA1c and FPG and percentages of patients at HbA1c goals of <7.0 and <6.5% after 24 weeks.

### Safety endpoints

Safety assessment included collection of adverse events, physical examination including vital signs, standard laboratory blood chemistry (e.g., liver and renal safety tests), lipid panel, hematology, urinalysis and electrocardiogram. A hypoglycemia log was provided to patients to collect hypoglycemia information. At the request of several regulatory authorities in the European Union, measurement of serum amylase and lipase were instituted after the study was initiated; therefore, not all patients had baseline assessments for these two measures.

Potential cases of pancreatitis (events assessed by the investigator as possibly being pancreatitis, or events meeting pre-specified event terms suggestive of pancreatitis) and pre-specified hypersensitivity adverse events (anaphylactic reaction, angioedema, asthma-bronchospasm, erythema multiforme, Stevens-Johnson syndrome, toxic epidermal necrolysis, and drug rash with eosinophilia and systemic symptoms) were evaluated in a blinded manner by external clinical adjudication committees.

### Statistical analyses

The population of all randomized patients who received at least one dose of study treatment and had a baseline or a post-randomization measurement served as the primary population for efficacy analyses.

For analysis of the primary efficacy endpoint, a longitudinal data analysis (LDA) model was used [6], with terms for treatment, time, sulfonylurea status at screening (treatment with glimepiride ≥4 mg/day at screening, treatment with glimepiride <4 mg/day at screening, and treatment with a sulfonylurea other than glimepiride at screening), the interaction of time by treatment and the interaction of time by sulfonylurea status at screening, with a constraint that the true mean at baseline is common to all treatment groups (which is valid due to randomization). The primary hypothesis regarding the superiority of omarigliptin over placebo in decreasing HbA1c was assessed using the estimated treatment difference from the LDA model.

Change from baseline in FPG at Week 24 was analyzed using the LDA model described above, substituting the appropriate baseline value.

Analysis of percentages of individuals at the HbA1c goals of <7.0% and <6.5% at Week 24 was based on estimated rates and confidence intervals for between-group rate differences computed using the Miettinen and Nurminen method [7]. Multiple imputations based on the LDA model used for the analysis of HbA1c were used to handle missing data [8]. Each patient was categorized as a responder (satisfying the HbA1c specific goal of <7.0 or <6.5%) or non-responder at Week 24.

Analysis of safety data used the population of all randomized patients who received at least one dose of study treatment. Safety and tolerability were assessed during the treatment period and through 21 days after treatment ended, by clinical review of all relevant parameters including adverse events, laboratory tests, electrocardiogram (ECG), vital signs and body weight. A *p*-value and the 95% CI for between-treatment difference in the percentage of subjects with adverse events of symptomatic hypoglycemia were calculated using the method of Miettinen and Nurminen [7]. For body weight, change from baseline was analyzed using the LDA model described above including terms for treatment, time, sulfonylurea status at screening, the interaction of time by treatment and the interaction of time by sulfonylurea status at screening. The 95% CI for between-treatment difference was calculated based on the estimate from the model. For adverse events with incidence of at least 4 patients in any treatment group, any adverse event of hypoglycemia and adverse events of severe hypoglycemia, 95% CIs were calculated for between-group comparisons using the method of Miettinen and Nurminen [7].

A sample size of approximately 300 patients randomized to omarigliptin or omarigliptin matching placebo in a 1:1 randomization ratio was expected to provide 135 patients per group for the analysis of mean change from baseline in HbA1c at Week 24. This sample size would provide 90% power to detect a true difference of 0.40% in the mean change from baseline in HbA1c between two treatment groups (two-sided test, α = 0.05). The half-width of the 95% CI was 0.24%.

## Results

### Patient disposition and characteristics

A total of 583 patients were screened and 307 were randomized (154 to omarigliptin and 153 to placebo). The most common reasons for a patient not being randomized were not meeting metformin and glimepiride dose requirements or meeting exclusionary laboratory values. The trial was initiated on 19-OCT-2012 and completed on 23-DEC-2014.

**Fig. 2:**
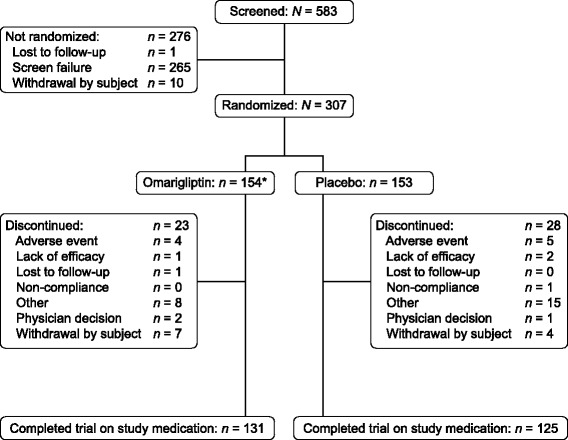
Patient disposition; *One subject in the omarigliptin group participated in two clinical trials, in parallel, at two different sites. The subject is included in this diagram but excluded from all efficacy and safety analyses

Of the 307 randomized patients, 256 (83.4%) completed the study on study medication (Fig. 2). One patient in the omarigliptin group who was discovered to be participating in another study was included in the population of randomized patients for the disposition table but excluded from the efficacy and safety analyses.

Baseline demographics and efficacy parameters were generally balanced between treatment groups (Table 1). The mean age was 57.8 years, approximately 52% were female, mean body mass index was 31.2 kg/m^2^, mean HbA1c of 8.6% and mean duration of diabetes was 10.1 years. Mean treatment compliance ± standard deviation for omarigliptin and placebo medication was 98.9% ± 3.5 and 98.3% ± 5.0, respectively.

**Table 1:** Baseline demographic, anthropometric and disease characteristics of study treatment groups

	Omarigliptin *N* = 154	Placebo *N* = 153
Age, years	57.2 ± 8.4	58.4 ± 9.4
Female, *n* (%)	81 (52.6)	79 (51.6)
Race, *n* (%)		
White	116 (75.3)	110 (71.9)
Asian	28 (18.2)	29 (19.0)
Black	8 (5.2)	7 (4.6)
Multi-racial	2 (1.3)	6 (3.9)
Native Hawaiian or other Pacific Island	0 (0.0)	1 (0.7)
Ethnicity, *n* (%)		
Not Hispanic or Latino	134 (87.0)	140 (91.5)
Hispanic or Latino	14 (9.1)	5 (3.3)
Not reported	2 (1.3)	3 (2.0)
Body Weight, kg	87.8 ± 18.3	85.4 ± 21.2
BMI, kg/m^2^	31.8 ± 6.2	30.6 ± 5.8
HbA1c, %	8.5 ± 0.8	8.6 ± 0.8
FPG^a^, mmol/L	10.1 ± 2.3	10.2 ± 2.3
Duration of type 2 diabetes, years	9.8 ± 5.3	10.4 ± 5.5
SU status at screening		
Glimepiride	103 (66.9)	104 (68.0)
SU other than glimepiride	51 (33.1)	49 (32.0)

Values are mean ± standard deviation unless otherwise noted

*BMI* body mass index, *FPG* fasting plasma glucose, *SU* sulfonylurea

^a^To convert to mg/dL multiply mmol/L value by 18

### Efficacy

**Fig. 3:**
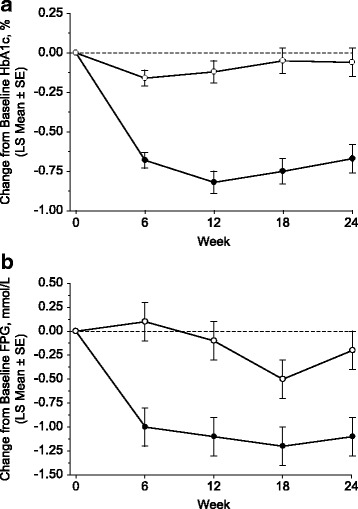
Efficacy measures through Week 24; **a** change from baseline HbA1c (%); **b** change from baseline FPG (mmol/L; to convert to mg/dL multiply mmol/L value by 18); ● omarigliptin, o placebo; based on the longitudinal data analysis model described in the Statistical Analyses Methods section

After 24 weeks of treatment, the least squares (LS) mean change from baseline in HbA1c (95% CI) was significantly greater with omarigliptin 25 mg q.w (−0.67% [−0.84, −0.50]) compared with placebo (−0.06% [−0.23, 0.12]) (Table 2 and Fig. 3a). The between-group difference (LS mean [95% CI]) in change from baseline at Week 24 in HbA1c was −0.61% (−0.85, −0.38); *p* < 0.001. A near maximum reduction in HbA1c was observed by Week 6 in the omarigliptin treatment group (Fig. 3a). Glycemic efficacy was maintained throughout the remainder of the treatment period.

**Table 2:** Efficacy endpoints at Week 24

Parameter	Omarigliptin *N* = 153	Placebo *N* = 153
HbA1c, %		
Baseline	8.5 ± 0.8	8.6 ± 0.8
Week 24	7.7 ± 1.1	8.4 ± 1.1
Change from baseline^a^	−0.67 (−0.84, −0.50)	−0.06 (−0.23, −0.12)
Change vs. placebo^b^	−0.61^d^ (−0.85, −0.38)	–
FPG^c^, mmol/L		
Baseline	10.2 ± 2.3	10.2 ± 2.3
Week 24	8.9 ± 2.2	9.8 ± 2.0
Change from baseline^a^	−1.1 (−1.5, −0.7)	−0.2 (−0.6, 0.2)
Change vs. placebo^b^	−0.9^d^ (−1.4, −0.4)	–

Values are mean ± standard deviation unless otherwise noted

^a^Least squares (LS) mean (95% CI)

^b^Difference in LS means (95% CI)

^c^To convert to mg/dL multiply mmol/L value by 18

^d^p < 0.001

After 24 weeks of treatment, the LS mean change from baseline in FPG was significantly greater in the omarigliptin group (−1.1 mmol/L) compared with the placebo group (−0.2 mmol/L). The between-group difference (LS mean [95% CI]) in change from baseline in FPG was −0.9 mmol/L (−1.4, −0.4); *p* < 0.001 (Table 2). A significant reduction in FPG in the omarigliptin group was observed by Week 6 (the first time point of measurement after randomization; Fig. 3b) and a treatment effect persisted throughout the treatment period.

The percentage (95% CI) of patients with an HbA1c *<*7*.*0% at week 24 was 23.8% (17.5, 31.5) in the omarigliptin group compared with 4.4% (2.1, 9.3) in the placebo group; between-group difference (95% CI) = 19.3 (11.7, 27.6); *p* < 0.001. The percentage (95% CI) of patients with HbA1c <6.5% at Week 24 was 10.1% (6.1, 16.4) in the omarigliptin group and 2.1% (0.7, 6.0) in the placebo group; between-group difference (95% CI) = 8.0 (2.7, 14.5); *p* = 0.005.

### Safety and tolerability

Summary measures of adverse events were generally similar between groups (Table 3). The percentage of patients with one or more adverse events was 57.5% in the omarigliptin group compared with 47.7% in the placebo group. No patients died during the study period.

**Table 3:** Adverse events (AEs) summary and AEs of hypoglycemia

Patients, *n* (%)	Omarigliptin *N* = 153	Placebo *N* = 153	Difference^a^
With one or more			
AEs	88 (57.5)	43 (47.7)	9.8 (−1.4, 20.8)
Drug-related^b^ AEs	12 (7.8)	10 (6.5)	1.3 (−4.8, 7.5)
Serious AEs	3 (2.0)	5 (3.3)	−1.3 (−5.7, 2.8)
Serious drug-related^b^ AEs	2 (1.3)	0 (0.0)	1.3
Who died	0 (0.0)	0 (0.0)	0.0
Who discontinued due to			
An AE	4 (2.6)	4 (2.6)	0.0 (−4.3, 4.3)
A drug-related^b^ AE	2 (1.3)	1 (0.7)	0.7
A serious AE	0 (0.0)	1 (0.7)	−0.7
A serious drug-related^b^ AE	0 (0.0)	0 (0.0)	0.0
With one or more AE of hypoglycemia	18 (11.8)	13 (8.5)	3.3 (−3.6, 10.3)
Symptomatic^c^	16 (10.5)	13 (8.5)	2.0^f^ (−4.8, 8.8)
Severe^d^	5 (3.3)	1 (0.7)	2.6 (−0.7, 6.9)
Asymptomatic^e^	2 (1.3)	0 (0.0)	1.3

^a^Difference in % vs placebo; estimate (95% CI) was computed only for AE summary with incidence of at least 4 patients in any treatment group, any adverse event of hypoglycemia and adverse events of severe hypoglycemia

^b^Assessed by the investigator as related to study drug

^c^Symptomatic hypoglycemia: episode with clinical symptoms attributed to hypoglycemia, without regard to glucose level

^d^Severe hypoglycemia: episode that required assistance, either medical or non-medical. Episodes with a markedly depressed level of consciousness, a loss of consciousness, or seizure were classified as having required medical assistance, whether or not medical assistance was obtained

^e^Asymptomatic hypoglycemia: fingerstick glucose values ≤3.9 mmol/L (70 mg/dL) without symptoms

^f^p = 0.559

The incidences of specific adverse events with an incidence ≥2% in one or more treatment group were generally similar between treatment groups (Table 4).

**Table 4:** Specific adverse events with an incidence ≥2% in one or more treatment group by system organ class

	Omarigliptin *N* = 153	Placebo *N* = 153
General disorders and administration site conditions		
Chest discomfort	3 (3.0)	0 (0.0)
Infections and infestations		
Influenza	3 (2.0)	0 (0.0)
Nasopharyngitis	4 (2.6)	5 (3.3)
Rhinitis	3 (2.0)	1 (0.7)
Upper respiratory tract infection	4 (2.6)	9 (5.9)
Urinary tract infection	9 (5.9)	3 (2.0)
Investigations		
Blood creatine phosphokinase increased	2 (1.3)	3 (2.0)
Blood glucose increased	1 (0.7)	3 (2.0)
Lipase increased	5 (3.3)	1 (0.7)
Metabolism and nutrition disorders		
Hyperglycemia	3 (2.0)	6 (3.9)
Hypoglycemia	18 (11.8)	13 (8.5)
Musculoskeletal and connective tissue disorders		
Arthralgia	3 (2.0)	3 (2.0)
Back pain	4 (2.6)	3 (2.0)
Nervous system disorders		
Headache	0 (0.0)	3 (2.0)

There was 1 case of adjudication-confirmed pancreatitis in the placebo group (and none in the omarigliptin group). One patient in the omarigliptin group had 1 non-serious adverse event of urticaria and 1 non-serious adverse event of angioedema, both of which were adjudicated and confirmed to be angioedema (a pre-specified hypersensitivity adverse event).

The incidences of patients with the adverse events of symptomatic hypoglycemia were 10.5% in the omarigliptin group and 8.5% in the placebo group (Table 3). The incidence of patients reported with severe hypoglycemia was 3.3% (5 patients) in the omarigliptin group compared with 0.7% (1 patient) in the placebo group (Table 3).

At Week 24, the change from baseline in body weight (LS mean [95% CI]) was −0. 1 kg [−0.7, 0.4] in the omarigliptin group and −0.9 [−1.4, −0.4] in the placebo group; between-group difference = 0.8 kg (0.1, 1.5).

From baseline to Week 24, there were no clinically meaningful changes in mean levels of chemistry analytes or clinically meaningful between-group differences in the incidence of patients meeting pre-defined limits of change for those analytes. Small increases from baseline at Week 24 in mean serum amylase and lipase levels were observed in both treatment groups. Both baseline and Week 24 mean amylase levels were within the normal range (35-121 U/L) in both treatment groups. Baseline mean serum lipase values were within the normal range (13-60 U/L) in both treatment groups, but at Week 24, mean serum lipase levels were slightly above the upper limit of the normal range in both treatment groups (75.7 IU/L ± 49.0 and 64.1 IU/L ± 53.8 in the omarigliptin and placebo groups, respectively). Increases in mean amylase and lipase levels were observed by Week 6 (the first measurement after baseline) and were non-progressive after that time point.

There were no clinically meaningful changes from baseline in pulse rate, blood pressure, or ECG intervals (including QTc) in either treatment group.

## Discussion

The present study demonstrated that in patients with T2D and inadequate glycemic control with the combination of metformin (≥1500 mg/day) and glimepiride (≥4 mg/day), treatment with omarigliptin 25 mg once-weekly was superior to placebo in achieving and maintaining glycemic control over 24 weeks. The magnitude of changes from baseline in HbA1c and FPG were consistent with those observed in similar add-on studies conducted with daily DPP-4 inhibitors, taking into account the differences in baseline HbA1c with those studies [9–11]. In addition, a numerically greater proportion of subjects in the omarigliptin group than in the placebo group met the HbA1c goals of <6.5% and <7.0%.

Omarigliptin was generally well tolerated. No clinically meaningful between-group differences in specific adverse events were observed. The incidence of hypoglycemia in both treatment groups is consistent with that observed in similar studies [9, 11, 12]. Due to their glucose-dependent mechanism of action, DPP-4 inhibitors are associated with low incidences of hypoglycemia when administered as monotherapy [13] or co-administered with agents that are not themselves associated with hypoglycemia [14, 15]; however, when they are administered with agents that are associated with hypoglycemia, such as sulfonylureas, the incidence of hypoglycemia is recognized to be increased [9, 11, 12]. Similar observations have been made with other antihyperglycemic agent classes which are not themselves associated with increased hypoglycemia [16, 17]. The mean changes from baseline in serum amylase and lipase, and reported adverse events related to these laboratory values, were not associated with any apparent clinical consequences in the omarigliptin group. The presence of baseline serum amylase and lipase values > ULN in approximately 10% of patients with T2D and the presence of mild asymptomatic elevations in amylase and lipase with the initiation of incretin treatment is a phenomena previously observed [18, 19].

Small decreases from baseline in body weight were observed in both treatment groups. The smaller weight loss in the omarigliptin group compared to placebo may be due to the improvement in glycemic control and attendant reduction in glycosuria-related calorie loss of the patients treated with omarigliptin. In omarigliptin studies that assessed omarigliptin as monotherapy and as add-on therapy with metformin, consistent decreases in body weight from baseline were observed in the omarigliptin groups [14, 20].

Previous studies have demonstrated the efficacy and safety of omarigliptin 25 mg q.w. as monotherapy and as an add-on to metformin for the treatment of T2D [19, 21, 22] and the present study extends these observations, providing evidence of the safety and efficacy of omarigliptin on a background of metformin and glimepiride.

## Conclusions

This study demonstrated that in patients with T2D and inadequate glycemic control on dual therapy with metformin and glimepiride, once-weekly omarigliptin compared with placebo provided greater improvement in glycemic control and was generally well-tolerated.

## Appendix 1

International Ethics Committees (IECs) by Country:

**Poland** Komisja Bioetyczna przy Okregowej Izbie Lekarskiej w Gdańsku, Gdansk 80-204 Poland. **Republic of Korea** Gachon University Gil Medical Center, Namdong-Gu Incheon 405-760 Republic of Korea; CHA Bundang Medical Center, CHA University, Seongnam-si, 487-010 Republic of Korea; EWHA Womans University Hospital, Seoul 158-710 Republic of Korea; Yonsei University College of Medicine, Seoul 120-752 Republic of Korea; The Catholic University of Korea, Seoul St. Mary’s Hospital, Seoul 137-701 Republic of Korea. **Romania** Comisia Naţională de Bioetică a Medicamentului şi a Dispozitivelor Medicale, Bucuresti 020125 Romania. **Russian Federation** Nizhny Novgorod Regional Clinic, Nizhny Novgorod 603,126 Russian Federation; Saratov State Medical University n.a. V.I. Razumovsky of Ministry of Health of Russia, Saratov 410,012 Russian Federation; Kazan State Medical University of Ministry of Health of Russia, Kazan, Republic of Tatarstan 420,043 Russian Federation; Expert Council on Biomedical Ethics under Bashkir State Medical University of Ministry of Health of Russia, Ufa 450,071 Russian Federation. **South Africa** Pharma-Ethics Independent Research Ethics Committee, Lyttelton Manor Gauteng 0157 South Africa. **United States** Schulman Associates Institution**,** Cincinnati, OH 45242 USA.

## Abbreviations

BMI: Body mass index
DPP-4: Dipeptidyl peptidase-4
ECG: Electrocardiogram
FPG: Fasting plasma glucose
LDA: Longitudinal data analysis
LS: Least squares
q.w.: Once-weekly
SU: Sulfonylurea
T2D: Type 2 diabetes
ULN: Upper limit of normal
